# Innovative Strategies in the Diagnosis and Treatment of Liver Cirrhosis and Associated Syndromes

**DOI:** 10.3390/life15050779

**Published:** 2025-05-13

**Authors:** Ashok Kumar Sah, Mohd Afzal, Rabab H. Elshaikh, Anass M. Abbas, Manar G. Shalabi, Pranav Kumar Prabhakar, Asaad M. A. Babker, Fariza Tursunbaevna Khalimova, Velilyaeva Aliya Sabrievna, Ranjay Kumar Choudhary

**Affiliations:** 1 Department of Medical Laboratory Sciences, College of Applied and Health Sciences, A’ Sharqiyah University, Ibra 400, Oman; rabab.mahmoud@asu.edu.om; 2 Department of Medical Laboratory Technology, Arogyam Institute of Paramedical & Allied Sciences (Affiliated to H.N.B. Uttarakhand Medical Education University), Roorkee 247661, India; afzalglocal@gmail.com; 3 Department of Clinical Laboratory Sciences, College of Applied Medical Sciences, Jouf University, Sakaka 72388, Saudi Arabia; anasseen@hotmail.com (A.M.A.); dr.mpathology@gmail.com (M.G.S.); 4 Department of Biotechnology, School of Engineering and Technology, Nagaland University, Meriema, Kohima 797004, India; prabhakar.iitm@gmail.com; 5 Department of Medical Laboratory Sciences, College of Health Sciences, Gulf Medical University, Ajman 4184, United Arab Emirates; azad.88@hotmail.com; 6 Department of Normal Physiology, Medical and Social Institute of Tajikistan, Dushanbe 734017, Tajikistan; 7 Department of Psychiatry, Medical Psychology, and Narcology, Samarkand State Medical University, Samarkand 140158, Uzbekistan; 8 Department of Medical Laboratory Technology, University Institute of Allied Health Sciences, Chandigarh University, Chandigarh 140413, India; 9 School of Paramedics and Allied Health Sciences, Centurion University of Technology and Management, Sitapur 761211, India

**Keywords:** liver cirrhosis, innovative diagnostics, targeted therapies, regenerative medicine, prognostic scoring

## Abstract

Liver cirrhosis continues to be a major global health issue, contributing to high morbidity and mortality due to its progressive nature and associated complications. This review explores recent advancements in the diagnosis and treatment of liver cirrhosis and its related syndromes. Non-invasive diagnostic tools, such as elastography and serum biomarkers, have significantly improved early detection, reducing the need for liver biopsies. Advanced imaging techniques, including MRI and CT, further enhance diagnostic accuracy. In parallel, molecular and genomic research is providing new insights into the pathogenesis of the disease, paving the way for precision medicine. On the treatment front, pharmacological innovations, such as antifibrotic agents and targeted therapies, show promise in slowing disease progression. Endoscopic interventions like variceal banding are improving the management of complications, while advancements in liver transplantation and artificial liver support systems offer life-saving alternatives. Regenerative medicine, particularly stem cell therapy and tissue engineering, is emerging as a promising strategy for liver repair. Managing cirrhosis-related syndromes, including portal hypertension, ascites, hepatic encephalopathy, and hepatorenal syndrome, now involves evolving therapeutic approaches such as transjugular intrahepatic portosystemic shunt (TIPS) and novel pharmacotherapies. Prognostic scoring systems like the MELD and Child–Pugh are being refined with new biomarkers for better risk stratification. The future of cirrhosis care will likely involve the integration of artificial intelligence and machine learning for early diagnosis and personalized treatments, alongside emerging therapies currently under investigation. Despite these advancements, challenges such as costs, accessibility, and healthcare disparities remain barriers to widespread adoption. This review highlights the importance of incorporating innovative diagnostic and therapeutic strategies into clinical practice to improve the outcomes for patients with liver cirrhosis and its complications.

## 1. Introduction

Liver cirrhosis is a major global health concern, characterized by the progressive replacement of healthy liver tissue with fibrotic scar tissue, leading to diminished liver function [1]. This condition results from various chronic liver diseases and is associated with high morbidity and mortality rates worldwide [2]. Cirrhosis represents the end stage of chronic liver disease, often caused by persistent liver damage from viral hepatitis, alcohol consumption, and non-alcoholic fatty liver disease [3,4]. Progression to cirrhosis signifies a severe decline in liver function, which, in some cases, can lead to life-threatening complications [5]. Liver cirrhosis and related chronic liver diseases pose a substantial global health burden [6]. Cirrhosis and other liver-related diseases were responsible for approximately 1.47 million deaths, highlighting their significant impact on global mortality rates [7]. The growing burden of chronic liver disease is primarily driven by the increasing prevalence of non-alcoholic fatty liver disease and alcohol-related liver disease [8].

This review aims to provide a comprehensive overview of recent advancements in the diagnosis and treatment of liver cirrhosis and its associated syndromes. By examining the latest developments and emerging therapies, we aim to highlight the evolving landscape of cirrhosis management and identify promising avenues for future research and clinical application.

## 2. Methodology

This review employed a comprehensive and systematic approach to identify and analyze the recent literature on novel diagnostic and therapeutic strategies for liver cirrhosis and its associated disorders. The literature search was conducted across multiple electronic databases, including PubMed, Scopus, Web of Science, and Google Scholar, focusing on papers published between January 2014 and March 2025. A combination of Medical Subject Headings (MeSH) terms and free-text keywords was used to ensure a thorough search. The key search terms included: “liver cirrhosis”, “diagnosis of liver cirrhosis”, “treatment of cirrhosis”, “innovative diagnostic strategies”, “novel therapies for cirrhosis”, “complications of cirrhosis”, “hepatocellular carcinoma”, “portal hypertension”, “liver fibrosis”, “non-invasive diagnostics”, and “emerging treatment modalities”. Boolean operators such as “AND”, “OR”, and “NOT” were applied to refine and broaden the search results as needed. This methodical approach ensured the inclusion of relevant and up-to-date studies for a comprehensive review of the current advancements in the field.

In addition to the database search, reference lists of chosen publications were manually reviewed to discover further relevant research that may have been overlooked during the first search. The retrieved articles were initially reviewed by title and abstract to determine their relevance to the subject of this review. Full-text copies of possibly suitable publications were then thoroughly reviewed in accordance with predetermined inclusion and exclusion criteria. The inclusion criteria required that the studies be published in English in peer-reviewed journals between 2014 and 2025, and that they focus specifically on advanced or innovative diagnostic techniques (such as imaging modalities, serological biomarkers, or elastography) or therapeutic strategies (including pharmacological, regenerative, or lifestyle interventions) related to liver cirrhosis and its syndromes. Both original research and review articles were considered, provided they presented substantial clinical or scientific insights.

Studies were excluded if they were published before 2014, written in a language other than English, or presented as editorials, letters, commentaries, conference abstracts, or case reports with limited relevance. Two independent reviewers conducted the screening and selection process. Any disagreements were resolved through discussion or, if necessary, consultation with a third reviewer to ensure the objectivity and quality of the review process.

## 3. Pathophysiology of Liver Cirrhosis

The final stage of chronic liver injury is liver cirrhosis, which is characterized by the gradual dysfunction of the liver caused by the replacement of healthy hepatic tissue with fibrotic scar tissue. Effective management requires knowledge of the underlying mechanisms, prevalent etiologies, and related problems [9,10].

**Figure 1:**
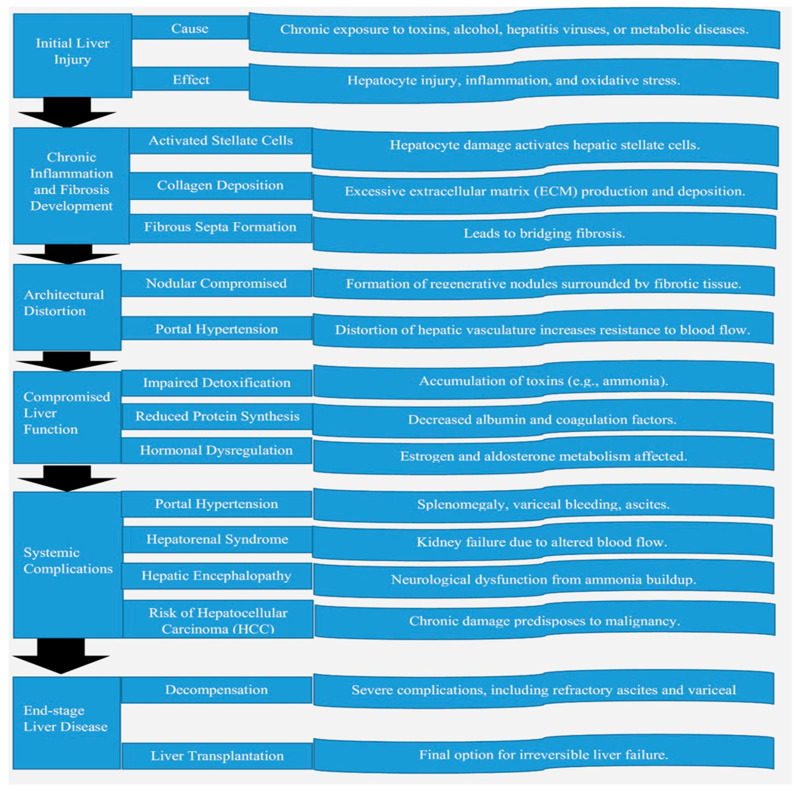
A structured outline detailing the sequential stages and underlying mechanisms driving the progression of liver cirrhosis, starting from early liver injury and culminating in advanced complications.

Figure 1 illustrates a systematic framework outlining the sequential pathophysiological stages and underlying molecular mechanisms involved in the progression of liver cirrhosis, beginning with initial hepatic injury and advancing toward end-stage complications.

### 3.1. Mechanisms Leading to Liver Cirrhosis

The pathogenesis of cirrhosis involves a complex interplay of cellular and molecular events [11,12].

Hepatocyte injury and death: Chronic liver shocks cause hepatocyte damage and apoptosis. DAMPs released by the dying cells activate hepatic stellate cells (HSCs) and Kupffer cells, the liver’s resident macrophages [13].

Activation of hepatic stellate cells (HSCs): HSCs store vitamin A during their dormant condition. When activated by inflammatory cytokines and oxidative stress, they develop into myofibroblast-like cells that produce an abundance of extracellular matrix components, resulting in fibrosis [14].

Fibrogenesis: The imbalance between fibrogenesis (scar tissue creation) and fibrolysis (scar tissue decay) promotes the buildup of fibrotic tissues. The activated HSCs release tissue inhibitors of metalloproteinases (TIMPs), which inhibit matrix metalloproteinases (MMPs), the enzymes that cause matrix breakdown [15,16].

Vascular alterations: Progressive fibrosis alters the hepatic vasculature, increasing the resistance to blood flow and leading to portal hypertension. Endothelial dysfunction and low nitric oxide bioavailability worsen intrahepatic vasoconstriction [17].

### 3.2. Common Etiologies of Liver Cirrhosis

Liver cirrhosis is the last stage of chronic liver disease, characterized by persistence and increasing liver damage [5]. Cirrhosis can be caused by a variety of factors, each with its own set of processes that lead to fibrosis, scarring, and liver failure [18]. Below is a thorough examination of the primary causes of liver cirrhosis.

#### 3.2.1. Viral Hepatitis

Chronic infections with hepatitis B virus (HBV) and hepatitis C virus (HCV) are the primary causes of liver cirrhosis worldwide. Persistent viral replication leads to ongoing inflammation and immune-mediated hepatocyte damage. This prolonged insult triggers a cycle of hepatocyte death, regeneration, and fibrogenesis [19].

Hepatitis B virus (HBV): HBV infection is distinguished by the incorporation of viral DNA into the host genome, which sustains inflammation even in dormant phases. Chronic HBV frequently causes cirrhosis and hepatocellular carcinoma (HCC) via pathways including oxidative stress and immune-mediated damage [20].

Hepatitis C virus (HCV): HCV-associated liver damage is caused by both the virus’s direct cytopathic effects and immune-mediated harm. Chronic HCV infection triggers fibrogenesis by secreting pro-inflammatory cytokines such as TNF-α and IL-6, which activate the hepatic stellate cells [21].

#### 3.2.2. Alcoholic Liver Disease (ALD)

Excessive and sustained alcohol intake is a leading cause of liver cirrhosis [22]. The pathogenesis of ALD includes the following:

Alcohol metabolism: In the liver, alcohol dehydrogenase (ADH) and cytochrome P450 2E1 (CYP2E1) metabolize ethanol to produce acetaldehyde, a highly reactive and poisonous metabolite. Acetaldehyde adducts with cellular proteins, causing oxidative stress and immunological activation [23].

Reactive oxygen species (ROS): The ROS produced during alcohol metabolism lead to lipid peroxidation, mitochondrial malfunction, and DNA damage [24].

***Inflammation:*** Alcohol increases intestinal permeability, resulting in endotoxemia and the activation of Kupffer cells, which produce inflammatory mediators that worsen liver damage [25].

Metabolic Dysfunction-Associated Steatohepatitis (MASH; formerly known as Non-Alcoholic Steatohepatitis, or NASH), a subtype of metabolic dysfunction-associated fatty liver disease (MAFLD), is distinguished by hepatic steatosis, inflammation, and fibrosis in the absence of heavy consumption of alcohol. It’s closely linked to metabolic syndrome, obesity, type 2 diabetes, and dyslipidemia [26]. Insulin resistance causes increased free fatty acid flux to the liver, resulting in steatosis. Oxidative stress and lipid peroxidation cause hepatocyte injury. Inflammatory mediators like TNF-α and IL-1β activate hepatic stellate cells, leading to fibrosis [27].

Autoimmune hepatitis (AIH): Autoimmune hepatitis is a chronic inflammatory disorder in which the immune system mistakenly attacks hepatocytes [28]. Autoimmune hepatitis (AIH) results from a combination of genetic predisposition and environmental stimuli, such as infections or drug exposure, that activate the autoreactive T cells [29]. These T cells actively assault the hepatocytes, causing ongoing inflammation and fibrosis. The histological features of AIH include lymphoplasmacytic infiltrates in the liver, interface hepatitis, and periportal fibrosis [30]. AIH advances to cirrhosis in around 40% of individuals who do not receive appropriate immunosuppressive medications, emphasizing the importance of early diagnosis and management [31].

***Cholestatic diseases:*** Cholestatic liver illnesses are characterized by reduced bile flow, which results in bile buildup and eventual liver damage [32].

***Primary biliary cholangitis (PBC):*** PBC is a chronic autoimmune illness marked by the loss of the intrahepatic bile ducts, resulting in cholestasis and cirrhosis. It is usually linked to antimitochondrial antibodies (AMAs) [33].

Primary sclerosing cholangitis (PSC): PSC is a progressive condition that causes inflammation and fibrosis of the intra- and extrahepatic bile ducts. PSC is significantly linked to inflammatory bowel disease (IBD) and an increased risk of cirrhosis and cholangiocarcinoma [34].

#### 3.2.3. Genetic Disorders

Several hereditary disorders cause liver damage by accumulating harmful chemicals. Hemochromatosis is caused by HFE gene mutations, which result in increased iron absorption and deposition in the liver, causing oxidative stress and fibrosis [35]. Similarly, Wilson’s Disease, a rare autosomal recessive condition caused by ATP7B gene mutations, causes copper to accumulate in hepatocytes [36]. The excess copper produces reactive oxygen species (ROS), which causes cellular damage and promotes fibrogenesis [37]. Another disorder, Alpha-1 Antitrypsin deficit, originates from a deficit of this protease inhibitor, leading to the buildup of misfolded proteins in the hepatocytes, which induces inflammation and fibrosis. Together, these genetic illnesses demonstrate the importance of inherited metabolic abnormalities in liver disease development [38].

## 4. Advances in Diagnosis of Liver Cirrhosis

The progressive nature of liver cirrhosis and its possibly deadly consequences make it a major global public health concern [39]. Effective care and better patient outcomes depend on early diagnosis. The diagnostic techniques have changed dramatically in recent years, with a focus on imaging, non-invasive techniques, and molecular/genomic procedures [40].

### 4.1. Non-Invasive Diagnostic Tools

FibroScan and elastography: FibroScan (transient elastography) and elastography are novel procedures that measure liver stiffness, a hallmark of fibrosis [41,42]. By providing quick, non-invasive substitutes for liver biopsies, these instruments lessen procedure hazards and patient pain. FibroScan measures liver stiffness using ultrasound-based elastography, yielding accurate findings in cases of alcohol-induced liver damage and hepatitis. Research shows that advanced cirrhosis and fibrosis can be diagnosed with high sensitivity and specificity; the accuracy increases when clinical markers are used [43,44].

Serum biomarkers: The APRI (AST-to-Platelet Ratio Index) and FIB-4 (Fibrosis-4 Index) are two non-invasive blood biomarkers that are now useful in the diagnosis of liver cirrhosis. Regular blood test results are used to compute these indicators, which provide affordable substitutes for invasive treatments. In environments with limited resources, the APRI is especially useful for differentiating between moderate fibrosis and advanced liver disease, whereas the FIB-4 combines the age, platelet count, ALT, and AST values for wider application [45,46].

### 4.2. Imaging Techniques

MRI, CT, and ultrasound advancements: Liver cirrhosis monitoring and diagnosis have greatly improved because of modern imaging methods, including MRI, CT, and ultrasound. Multiphasic CT and contrast-enhanced MRI provide precise visualizations of the liver parenchyma and vascular alterations linked to cirrhosis. By evaluating the tissue diffusion properties, methods such as diffusion-weighted MRI (DW-MRI) allow for the assessment of liver fibrosis [47]. Additionally, the real-time, non-invasive evaluation of liver stiffness and perfusion problems is made possible by developments in ultrasound, such as contrast-enhanced ultrasonography (CEUS) and shear wave elastography (SWE) [48].

### 4.3. Molecular and Genomic Approaches

Role of genetic markers and molecular diagnostics: Recent advancements in molecular and genomic technologies have significantly improved the accuracy of liver cirrhosis diagnosis [49]. Genetic markers, such as single nucleotide polymorphisms (SNPs) in genes like PNPLA3 and TM6SF2, have been linked to an increased risk of liver fibrosis and cirrhosis [50]. Molecular diagnostics, including the analysis of circulating cell-free DNA (cfDNA) and microRNAs (miRNAs), have provided deeper insights into liver damage, inflammation, and fibrosis at the molecular level [51]. These biomarkers not only aid in diagnosing liver cirrhosis, but also help to predict disease progression and responses to treatment. For instance, miR-122, a liver-specific miRNA, has shown promise as a non-invasive biomarker for detecting liver damage, making it a potential tool for monitoring disease activity and therapeutic outcomes [52]. Genomic studies using next-generation sequencing (NGS) have uncovered novel fibrogenesis pathways, offering up new possibilities for targeted therapy. Epigenetic alterations, such as DNA methylation patterns in genes like RASSF1A, have diagnostic value [53,54].

Table 1 underscores the evolving landscape of liver cirrhosis diagnostics, emphasizing the clinical potential of integrating conventional methods with emerging technologies to enhance diagnostic accuracy and patient management.

## 5. Innovative Treatment Modalities for Liver Cirrhosis

The introduction of novel therapeutic techniques has transformed the management of liver cirrhosis [47]. These treatments seek to slow disease development, reduce complications, and increase survival rates. This section discusses the significant areas of advancement, such as pharmaceutical therapy, endoscopic procedures, transplantation breakthroughs, and regenerative medicines [64].

### 5.1. Pharmacological Therapies

***Antifibrotic agents:*** Antifibrotic therapies are emerging as a promising approach for the treatment of liver cirrhosis [65]. These agents target the key processes in fibrosis, including TGF-β signaling, hepatic stellate cell (HSC) activation, and extracellular matrix deposition [66]. Drugs like pirfenidone and simtuzumab have shown potential in both preclinical and clinical studies for treating liver fibrosis [67]. In addition, inhibitors of lysyl oxidase-like 2 (LOXL2) and integrins are currently being investigated for their antifibrotic effects in cirrhosis [68].

***Targeted therapies for specific etiologies:*** Addressing the root causes of cirrhosis is crucial for effective treatment [69]. In the case of viral hepatitis-induced cirrhosis, antiviral therapies such as direct-acting antivirals (DAAs) for hepatitis C and nucleos(t)ide analogs for hepatitis B have proven successful in slowing the disease progression and, in some cases, even reversing fibrosis in the early stages [70]. Additionally, the emerging treatments for metabolic dysfunction-associated fatty liver disease (MAFLD), including GLP-1 agonists and SGLT2 inhibitors, show significant promise for improving outcomes in these patients [71].

### 5.2. Endoscopic Interventions

Variceal banding and sclerotherapy: Endoscopic procedures are necessary for addressing cirrhosis complications such as esophageal varices [72]. Variceal band ligation (VBL) is the most effective treatment for variceal bleeding, with fewer problems than sclerotherapy [73]. Endoscopic sclerotherapy, while less commonly utilized, is nonetheless useful in resource-constrained situations. Combining these procedures with pharmaceutical treatments, such as non-selective beta-blockers, lowers the chance of rebleeding and death [74].

### 5.3. Transplantation Advances

Innovations in liver transplantation techniques: End-stage liver cirrhosis is treated definitively with a liver transplant. Recent developments such as normothermic machine perfusion (NMP) have increased graft preservation and donor organ survival [75]. The split-liver and living-donor transplantation procedures have increased the donor pool, therefore meeting the growing need for transplants. Furthermore, advances in immunosuppressive medications are lowering transplant rejection and increasing long-term survival [76].

Artificial liver support systems: Artificial liver support systems, including bioartificial livers and extracorporeal liver assistance devices (ELADs), are being developed to bridge the gap between transplantation and acute decompensation recovery [77]. These systems use bioreactors containing hepatocytes to conduct detoxifying and synthesis tasks, possibly reducing the requirement for transplantation [78].

### 5.4. Regenerative Medicines

Stem cell therapy and tissue engineering: Regenerative medicine has novel possibilities for curing cirrhosis. Mesenchymal stem cells (MSCs) have showed promise in decreasing fibrosis and enhancing liver regeneration through immune response modulation and growth factor secretion [79,80]. Clinical research on bone marrow-derived and umbilical cord-derived MSCs has shown their safety and potential effectiveness [81].

Tissue engineering technologies, such as the 3D bioprinting of liver tissue and decellularized liver scaffolds, are still in the early phases of research. These methods seek to generate functioning liver tissues for transplantation or study, perhaps alleviating organ shortages [82].

Table 2 presents advanced therapeutic strategies for liver cirrhosis, highlighting the contributions of technological innovations, precision medicine, and regenerative therapies in optimizing clinical outcomes and enhancing patient care.

## 6. Managing Associated Syndromes of Liver Cirrhosis

Liver cirrhosis is frequently associated with severe symptoms such as portal hypertension, ascites, hepatic encephalopathy, and hepatorenal syndrome [96]. The treatment of these illnesses has changed dramatically as new therapeutic techniques have emerged. The following is a thorough review of the developments in controlling these issues [97].

### 6.1. Portal Hypertension

A significant consequence of cirrhosis is portal hypertension, which is caused by an increased resistance to portal blood flow. It is diagnosed with both invasive technologies like HVPG (Hepatic Venous Pressure Gradient) and non-invasive instruments like Doppler ultrasonography and elastography. Non-selective beta-blockers, endoscopic band ligation, and TIPS are used to treat refractory patients. Recent imaging breakthroughs help to monitor and forecast the results. A clear diagnostic-to-therapeutic flow is critical for successful treatment [98].

TIPS (Transjugular Intrahepatic Portosystemic Shunt): TIPS includes employing stents to create a low-resistance route between the portal and systemic venous systems, which reduces the portal pressure and alleviates problems such as variceal hemorrhage and refractory ascites [99]. Recent improvements include coated stents, which have increased patency rates and reduced problems such as hepatic encephalopathy [100].

According to studies, TIPS can be given proactively in high-risk variceal hemorrhage episodes, increasing survival results. Furthermore, advanced imaging modalities like intravascular ultrasonography (IVUS) improve the procedure’s accuracy [101,102].

### 6.2. Ascites

Cirrhosis-related portal hypertension is frequently accompanied by ascites, or fluid buildup in the peritoneal cavity [103].

Advances in diuretics: Combination diuretic therapy with spironolactone and furosemide is the primary treatment for ascites [104]. Advances in personalized medicine, such as renal function monitoring, are helping to optimize the dose and reduce adverse effects. New medicines targeting aquaporin channels and vasopressin V2 receptor antagonists, such as tolvaptan, show promise in treating refractory ascites [105].

Paracentesis techniques: Large-volume paracentesis (LVP) is the first-line treatment for refractory ascites. Safety innovations include ultrasound-guided methods and the use of albumin infusion to minimize paracentesis-induced circulatory dysfunction (PICD). Portable, less invasive methods for ascites drainage, such as automated paracentesis pumps, are being tested to increase patient convenience [106].

### 6.3. Hepatic Encephalopathy

Hepatic encephalopathy (HE) is a neuropsychiatric condition resulting from a buildup of ammonia and other neurotoxins [107].

New therapeutic strategies: Rifaximin, a non-absorbable antibiotic, has become the standard in HE therapies, especially when combined with lactulose [108]. Recent research reveals that probiotics and synbiotics can help lower ammonia production and improve cognitive symptoms [109].

Clinical trials are underway for emerging medicines that target the ammonia metabolism, including ornithine phenylacetate and glycerol phenylbutyrate, as well as innovative approaches for severe HE, such as extracorporeal ammonia removal utilizing dialysis-like equipment [110].

### 6.4. Hepatorenal Syndrome

Hepatorenal syndrome (HRS) is a serious consequence caused by the renal failure in advanced cirrhosis [111]. Type 1 HRS is now referred to as HRS-AKI (Hepatorenal Syndrome–Acute Kidney Injury), whilst a more chronic type is designated as HRS-CKD. Emerging treatment options: Therapies that combine vasoconstrictors (e.g., terlipressin) with albumin infusion remain the foundation for HRS-AKI therapy. Terlipressin has been shown in recent trials to minimize renal impairment and enhance survival in HRS-AKI, although it carries a risk of ischemic consequences [112]. Emerging medicines such as angiotensin II inhibitors, endothelin receptor antagonists, and prostaglandin analogs are being tested. Advanced renal replacement treatments, such as sustained low-efficiency dialysis (SLED) and continuous renal replacement therapy (CRRT), are increasingly being employed in acute situations [113].

## 7. Role of Prognostic Scoring in Liver Cirrhosis

Prognostic scoring systems are critical for determining the severity of liver cirrhosis, anticipating complications, making treatment decisions, and calculating survival. Over time, many scoring methods have evolved, with newer models including sophisticated biomarkers to improve the prognostic accuracy [114,115].

### 7.1. Scoring Systems and Integration of New Biomarkers

MELD Score (Model for End-Stage Liver Disease): Initially developed to predict mortality after transjugular intrahepatic portosystemic shunt (TIPS), the MELD score is now widely used for liver transplantation prioritization. It is calculated using three key laboratory parameters: serum bilirubin (indicating the liver excretory function), serum creatinine (reflecting the renal dysfunction), and the INR (International Normalized Ratio) (assessing the synthetic liver function). The MELD formula is given in [116]:

MELD = 10 × (0.957 × ln[creatinine] + 0.378 × ln[bilirubin] + 1.120 × ln[INR] + 0.643)

Child–Pugh Score: One of the earliest predictive tools for cirrhosis, the Child–Pugh score categorizes patients into three classes (A, B, and C) based on disease severity. It evaluates bilirubin, albumin, prothrombin time/INR, ascites, and hepatic encephalopathy, with each parameter scored from 1 to 3. Although simple and widely used, it is less precise than the MELD in predicting the short-term mortality [117].

### 7.2. Integration of New Biomarkers into Prognostic Models

Role of biomarkers: The integration of novel biomarkers aims to enhance the accuracy of prognostic models like the MELD and Child–Pugh by addressing their limitations [118]. The key biomarkers under investigation include C-reactive proteins (CRPs), which indicate systemic inflammation and are linked to a poor cirrhosis prognosis; fibrinogen, a marker of coagulopathy and systemic inflammation; alpha-fetoprotein (AFP), which may signal hepatocellular carcinoma or advanced liver disease; and soluble CD163, a macrophage activation marker associated with portal hypertension and liver inflammation. Incorporating these biomarkers can improve the predictive precision of the existing models [119,120].

Genomic and molecular markers: Emerging genomic and molecular markers are being incorporated into advanced prognostic models to improve disease prediction. Genetic polymorphisms such as PNPLA3 and TM6SF2 are associated with disease progression and fibrosis severity; however, such testing is mainly considered for select high-risk populations, rather than for all cirrhotic patients [121]. MicroRNAs (miRNAs), including miR-122 and miR-29a, are being studied for their potential to predict fibrosis and cirrhosis outcomes. Additionally, proteomics-based markers such as TIMP-1 and PIIINP, which are involved in extracellular matrix remodeling, serve as indicators of liver fibrosis progression. Integrating these biomarkers enhances the precision of liver disease assessments [122].

MELD 3.0 and other evolving models: MELD 3.0 includes additional indicators such as serum albumin and sex-specific modifications, which improves the predicted accuracy for post-transplant death. Furthermore, composite indices that include biomarkers and clinical assessments (such as FibroMeter and HepaScore) are being investigated to improve prognosis [123].

## 8. Future Directions in Liver Cirrhosis Management

The treatment of liver cirrhosis is evolving rapidly, driven by technological advancements, innovative therapies, and the growing potential of precision medicine. The integration of artificial intelligence (AI), machine learning (ML), and emerging therapeutic strategies holds immense promise for enabling early detection, personalized treatment plans, and improved patient outcomes [124]. These advancements are paving the way for more accurate diagnoses and more effective, tailored interventions, offering new hope for patients with cirrhosis.

### 8.1. Role of AI and Machine Learning in Early Diagnosis

Artificial intelligence and machine learning are transforming healthcare, especially in the early diagnosis of liver cirrhosis. These technologies use enormous datasets to uncover patterns that are not visible using the standard diagnostic approaches [125].

#### Applications in Liver Cirrhosis Diagnosis

Imaging analysis: AI-powered systems are rapidly being utilized to analyze various imaging modalities, such as ultrasound, CT, and MRI. They improve the diagnostic accuracy by identifying small variations in liver structure and fibrosis. For example, convolutional neural networks (CNNs) can accurately detect early-stage cirrhosis [126].

Risk prediction models: ML algorithms provide risk models for disease development by combining clinical data, imaging, and biomarkers. For example, models based on longitudinal data can forecast the shift from compensated to decompensated cirrhosis [127].

Natural language processing (NLP): NLP approaches applied to electronic health records (EHRs) assist in identifying undetected or misdiagnosed instances of cirrhosis by extracting crucial clinical information [128].

### 8.2. Precision Medicine Approaches

Precision medicine customizes therapies for each patient based on genetic, molecular, and environmental characteristics. This approach to liver cirrhosis tries to maximize the treatment efficacy while minimizing unwanted effects [129].

#### Key Developments in Precision Medicine

Genomic profiling: Advances in genome sequencing have enabled the discovery of genetic variants (e.g., PNPLA3, TM6SF2) linked to cirrhosis risk and progression. Such profiling helps to stratify patients and identify relevant therapies [130].

Molecular targeting: Therapies are being developed to target particular fibrosis pathways, such as TGF-β and Hedgehog signaling. Biomarker-driven selection guarantees the success of these medicines in specified patient groupings [131].

Pharmacogenomics: Pharmacogenomic studies can assist in optimizing medication doses and preventing adverse responses in cirrhotic patients. For example, genetic testing for CYP2D6 variations can help guide beta-blocker treatments for portal hypertension [132].

### 8.3. Emerging Therapies Under Clinical Trials: Therapies Targeting Fibrosis

Galectin-3 inhibitors: Drugs like belapectin are being tested in clinical studies to slow fibrosis development by blocking galectin-3, a critical mediator in liver fibrogenesis [133].

Anti-LOXL2 therapies: Lysyl oxidase-like 2 (LOXL2) inhibitors are being investigated for their ability to diminish extracellular matrix remodeling in cirrhosis [134].

Immunotherapies: Immunomodulatory drugs, such as checkpoint inhibitors, are being studied for their ability to reduce fibrosis while also treating hepatocellular cancer [135].

Stem cell therapies: Mesenchymal stem cell (MSC) transplantation has promise for rebuilding damaged liver tissues and regulating immune responses in cirrhotic individuals [136].

## 9. Challenges in Implementing Innovative Strategies for Liver Cirrhosis

Despite the substantial advances in the diagnosis and treatment of liver cirrhosis, various barriers prevent the broad use of novel techniques. These issues include access and cost constraints, limits in the existing diagnostic and treatment technologies, and global healthcare inequities. Accessibility and cost barriers: Innovative technologies and medicines are sometimes expensive, making them inaccessible in low-resource situations. These constraints are most visible in underdeveloped nations, where healthcare expenditures are limited and health insurance coverage is insufficient.

### 9.1. Costs of Advanced Diagnostics and Therapies

Diagnostic tools: Transient elastography (FibroScan) and sophisticated imaging modalities (MRI, CT) are costly, making them unavailable in rural and underdeveloped areas.

*Therapeutics:* Novel antifibrotic medicines, stem cell treatments, and liver transplantation remain unavailable to the majority due to their high costs and restricted availability in healthcare systems with constrained resources.

*Infrastructure gaps:* Shortages of specialized diagnostic equipment and skilled staff exacerbate the accessibility issue in rural healthcare institutions.

### 9.2. Limitations of Current Diagnostic and Therapeutic Approaches

While the advances in diagnostic and therapeutic technologies are promising, they are not without limitations.

#### 9.2.1. Diagnostic Limitations

Accuracy and reliability: Non-invasive diagnostics, such as APRI, FIB-4, and FibroScan, have varied sensitivities and specificities for the different types of liver disease. Their performances may be unsatisfactory in individuals with comorbidities such as obesity or ascites.

Early detection: The existing diagnostic techniques frequently fail to detect cirrhosis in its early stages, when therapies may be more beneficial. This constraint emphasizes the importance of more sensitive biomarkers and imaging approaches.

#### 9.2.2. Therapeutic Limitations

Efficacy: Despite the advancements, antifibrotic medications are not curative, and are largely intended to halt disease progression. Furthermore, several of the medicines in clinical trials have yet to show long-term success in varied patient groups.

Adverse effects: Some medicines, such as immunomodulatory and stem cell treatments, carry considerable risks and problems, restricting their broad usage.

### 9.3. Addressing Disparities in Global Healthcare

Healthcare disparities offer substantial barriers for the application of new liver cirrhosis treatment techniques, particularly in low- and middle-income countries.

#### Global Disparities

Resource distribution: Advanced diagnostic and treatment technologies are mostly found in metropolitan tertiary care facilities, leaving the rural and marginalized populations underserved. Awareness and Education: A lack of understanding of liver cirrhosis among patients and primary care providers contributes to delayed diagnosis and treatment, especially in resource-constrained areas.

Policy and funding gaps: The inadequate financing for public health initiatives and infrastructure development worsens healthcare inequities. Furthermore, patients in low- and middle-income countries continue to bear large out-of-pocket payments.

## 10. Conclusions

The landscape of liver cirrhosis diagnosis and treatment has been transformed by significant advancements in medical technologies and therapeutic strategies. Non-invasive diagnostic tools such as elastography and serum biomarkers have improved early detection, reducing the reliance on invasive procedures like liver biopsy. Additionally, precision imaging techniques, including advanced MRI and CT scans, have enhanced diagnostic accuracy, while molecular and genomic research has paved the way for precision medicine, offering more tailored treatment approaches. On the therapeutic front, targeted pharmacological treatments such as antifibrotic agents show promise in slowing disease progression. Endoscopic and interventional procedures, including variceal banding and transjugular intrahepatic portosystemic shunt (TIPS), have significantly improved the management of cirrhosis-related complications. Regenerative medicines, particularly stem cell therapy and tissue engineering, represent a groundbreaking frontier in liver repair. Furthermore, artificial intelligence (AI) and machine learning are revolutionizing early disease detection, risk stratification, and personalized treatment strategies, enabling more efficient and effective patient care. However, despite these remarkable advancements, several challenges remain. The limited accessibility, high costs, and disparities in healthcare systems hinder the widespread adoption of these innovations. Overcoming these barriers requires continued research, policy reforms, and international collaboration to ensure equitable access to state-of-the-art diagnostic and therapeutic solutions.

Further, a multidisciplinary approach integrating innovative diagnostic tools, novel treatments, and AI-driven solutions will be critical in optimizing liver cirrhosis management. By addressing the existing gaps and leveraging technological advancements, healthcare systems can enhance patient outcomes and improve the quality of life for individuals affected by liver cirrhosis worldwide.

## Figures and Tables

**Table 1:** This table highlights the evolving landscape of liver cirrhosis diagnostics, emphasizing the potential of combining traditional and innovative techniques for improved patient care.

Ref.	Category	Technique	Description	Advantages	Limitations
[55]	**Non-Invasive Diagnostic Tools**	FibroScan (Transient Elastography)	Measures liver stiffness non-invasively to assess fibrosis.	Quick, painless, portable, and widely used.	Limited accuracy in patients with obesity or significant ascites.
[56]		Shear Wave Elastography	Advanced ultrasound-based technology to measure liver stiffness.	High resolution and accuracy.	Operator dependency and higher cost.
[57]		Serum Biomarkers (e.g., APRI, FIB-4)	Blood tests calculating scores based on liver enzymes and platelet counts.	Non-invasive, inexpensive, and widely available.	Limited specificity and sensitivity in early-stage cirrhosis.
[58]	**Imaging Techniques**	Ultrasound	Common imaging for liver assessment and detection of nodular patterns.	Widely accessible and cost-effective.	Limited in detecting mild fibrosis.
[59]		Advanced MRI (e.g., MRE, DWI-MRI)	Magnetic resonance elastography (MRE) and diffusion-weighted imaging (DWI) for detailed fibrosis mapping.	Superior sensitivity and specificity for detecting fibrosis and inflammation.	High cost and limited availability.
[60]		CT Imaging	Provides detailed liver architecture and identifies complications like varices.	Effective for detecting advanced cirrhosis and complications.	Involves radiation exposure; limited use in early-stage diagnosis.
[61]	**Molecular and Genomic Approaches**	Genetic Markers	Identification of mutations and genetic predisposition for liver diseases.	Enables personalized risk assessment and targeted therapies.	Requires advanced laboratory facilities and high costs.
[62]		Molecular Diagnostics (e.g., miRNA)	Detection of specific biomarkers, such as microRNAs, linked to fibrosis and inflammation.	High accuracy in early-stage diagnosis and progression monitoring.	Requires specialized expertise and equipment.
[63]		Multiomics Approaches	Integration of genomics, proteomics, and metabolomics for comprehensive liver disease profiling.	Holistic understanding of disease pathways and potential therapeutic targets.	Complexity, high cost, and limited widespread application.

**Table 2:** This table shows cutting-edge treatment options for liver cirrhosis, emphasizing the roles of technology, precision medicine, and regenerative approaches in improving patient outcomes.

Ref.	Category	Treatment Modality	Description	Advantages	Challenges/Concerns
[83]	Pharmacological Therapies	Antifibrotic Agents	Drugs targeting fibrogenesis pathways to reduce liver scarring (e.g., simtuzumab, losartan).	Slows or reverses fibrosis progression.	Limited effectiveness in late-stage illness, possible side effects, lack of licensed medications, continuing clinical trials, and regulatory ambiguity.
[84]		Targeted Therapies	Antivirals for hepatitis (e.g., tenofovir, entecavir), immunotherapies for autoimmune etiologies.	Treats underlying causes of liver disease.	Contraindications in certain comorbidities, unpleasant effects, high costs, little clinical evidence, ethical problems in long-term usage, and pending regulatory clearance.
[85]		Gut Microbiota Modulation	Probiotics or fecal microbiota transplantation (FMT) to restore gut–liver axis health.	Improves inflammation and reduces endotoxemia.	Variable patient response, danger of infection, ethical problems with FMT, lack of standardization, and insufficient regulatory advice.
[86]	Endoscopic Interventions	Variceal Banding	Bands placed on esophageal varices to prevent bleeding.	Minimally invasive and effective for variceal bleeding prevention.	Requires competent staff; danger of ulceration and rebleeding; may require several sessions; contraindicated in active infections or recalcitrant individuals.
[87]		Sclerotherapy	Injection of sclerosants into varices to control bleeding.	Immediate bleeding control.	High risk of complications (e.g., ulceration, perforation), contraindicated in severe coagulopathy, may induce recurrence, and less recommended than banding owing to side effects.
[88]	Transplantation Advances	Innovations in Liver Transplantation	Use of marginal donors, split-liver transplantation, and robotic surgery.	Expands donor pool and improves surgical precision.	Living donation raises ethical difficulties because of its high cost, danger of rejection, long-term immunosuppressive effects, and restricted availability in resource-poor regions.
[89]		Artificial Liver Support Systems	Bioartificial livers and extracorporeal liver support systems (e.g., MARS).	Provides temporary support for acute liver failure.	High costs, limited availability, lack of long-term effectiveness, contraindications in multi-organ failure, and no permanent cure.
[90]	Regenerative Medicine	Stem Cell Therapy	Use of mesenchymal stem cells to regenerate damaged liver tissue.	Potential to repair liver damage and delay transplantation.	Lack of large-scale clinical studies, regulatory limits, potential immunological responses, ethical problems, and uncertainty about long-term safety and efficacy.
[91]		Tissue Engineering	Creation of bioengineered liver tissue for transplantation.	Addresses organ shortage crisis.	Mostly preclinical, ethical and regulatory difficulties, high expenses, technological complexity, and lack of functional long-term results in people.
[92]		Gene Therapy	Modifying genes to prevent or treat liver fibrosis and cirrhosis.	Potential for curative treatment of genetic liver diseases.	High cost, safety issues (e.g., immunological responses, off-target effects), ethical difficulties, little clinical evidence, and complicated regulatory approval.
[93]	Emerging Approaches	AI-Guided Personalized Therapies	AI-driven algorithms to design individualized treatment plans.	Enhances treatment precision and efficiency.	Data privacy problems, reliance on high-quality data, possible bias in algorithms, legal barriers, and inadequate long-term clinical validation.
[94]		RNA-Based Therapies	Use of RNA interference (RNAi) to silence fibrotic genes (e.g., siRNA drugs).	Targets fibrosis at molecular level.	Limited clinical evidence, possible off-target effects, immunological responses, high costs, and regulatory issues.
[95]		Nano-Drug Delivery Systems	Nanoparticles for targeted delivery of antifibrotic drugs.	Reduces off-target effects and enhances drug efficacy.	Limited clinical evidence, possible toxicity of nanoparticles, high cost, regulatory barriers, and scalability issues.
